# Heterologous Naringenin Production in the Filamentous Fungus *Penicillium rubens*

**DOI:** 10.1021/acs.jafc.3c06755

**Published:** 2023-12-16

**Authors:** Bo Peng, Lin Dai, Riccardo Iacovelli, Arnold J. M. Driessen, Kristina Haslinger

**Affiliations:** † Chemical and Pharmaceutical Biology, Groningen Research Institute of Pharmacy, University of Groningen, Antonius Deusinglaan 1, 9713AV Groningen, The Netherlands; ‡ Molecular Microbiology, Groningen Biomolecular Sciences and Biotechnology Institute, University of Groningen, Nijenborgh 7, 9747AG Groningen, The Netherlands

**Keywords:** flavonoids, polyketides, biotransformation, biosynthesis, pathway engineering

## Abstract

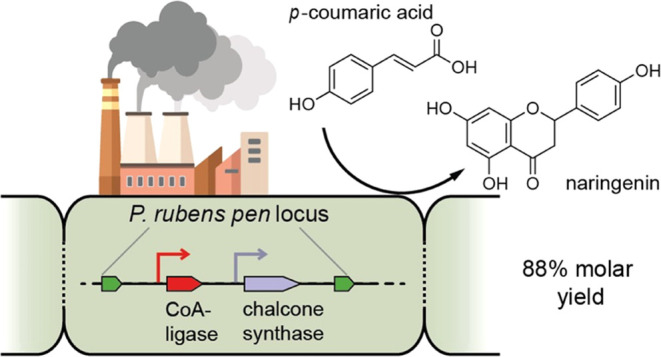

Naringenin is a natural product with several reported bioactivities and is the key intermediate for the entire class of plant flavonoids. The translation of flavonoids into modern medicine as pure compounds is often hampered by their low abundance in nature and their difficult chemical synthesis. Here, we investigated the possibility to use the filamentous fungus *Penicillium rubens* as a host for flavonoid production. *P. rubens* is a well-characterized, highly engineered, traditional “workhorse” for the production of β-lactam antibiotics. We integrated two plant genes encoding enzymes in the naringenin biosynthesis pathway into the genome of the secondary metabolite-deficient *P. rubens* 4xKO strain. After optimization of the fermentation conditions, we obtained an excellent molar yield of naringenin from fed *p-*coumaric acid (88%) with a titer of 0.88 mM. Along with product accumulation over 36 h, however, we also observed rapid degradation of naringenin. Based on high-resolution mass spectrometry analysis, we propose a naringenin degradation pathway in *P. rubens* 4xKO, which is distinct from other flavonoid-converting pathways reported in fungi. Our work demonstrates that *P. rubens* is a promising host for recombinant flavonoid production, and it represents an interesting starting point for further investigation into the utilization of plant biomass by filamentous fungi.

## 1 Introduction

**Figure 1:**
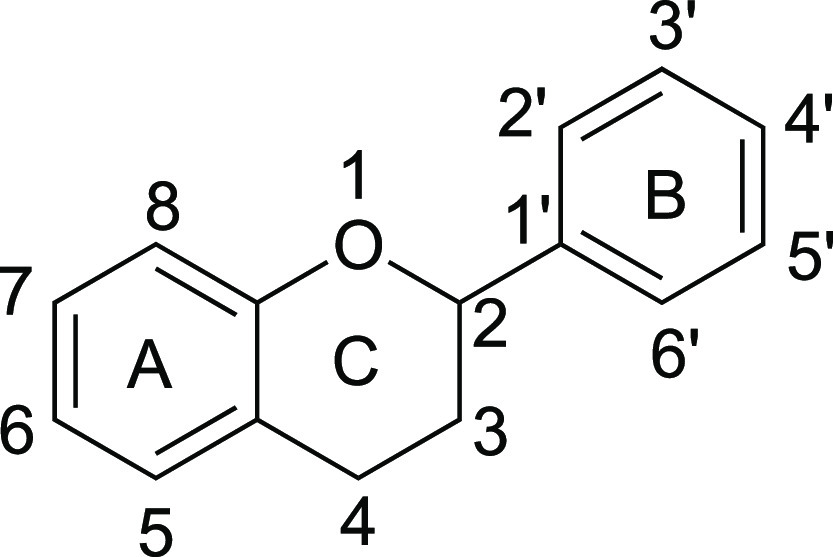
Basic structure of flavonoids.

Flavonoids are natural products found in various fruits, vegetables, and flowers. They belong to a class of plant secondary metabolites with a polyphenolic structure (Figure 1). Plants use flavonoids for the growth and development of seedlings, the production of color and aromas to attract pollinators, and to protect themselves against different biotic and abiotic stresses.^1,2^ For humans, flavonoids are an integral part of our diet and are mostly responsible for the color, taste, prevention of fat oxidation, and protection of vitamins and enzymes in food.^3,4^ Additionally, flavonoids are reported to have several benefits for human health. This has been attributed to their antioxidant, antitumor, antiviral, anti-inflammatory, and neuroprotective activities, which have been reported in experiments with mammalian cell cultures.^5−7^ These health-promoting effects make flavonoids highly attractive for nutraceutical, pharmaceutical, and cosmetic applications.^2^

Unfortunately, the current manufacturing routes do not provide scalable processes for large-scale production of pure flavonoids, hampering the study of these molecules for wider applications. The various extraction and purification steps needed for isolation from plant material, or the chemical synthesis come at high production costs and negatively impact the environment.^8^ This is due to the low relative abundance of flavonoids in the plant tissues and the difficulty in separating them on a preparative scale since they exist as complex mixtures of structurally similar compounds. Furthermore, the cultivation of plants for flavonoid extraction is rather inefficient because of long growing seasons.^8^

**Figure 2:**
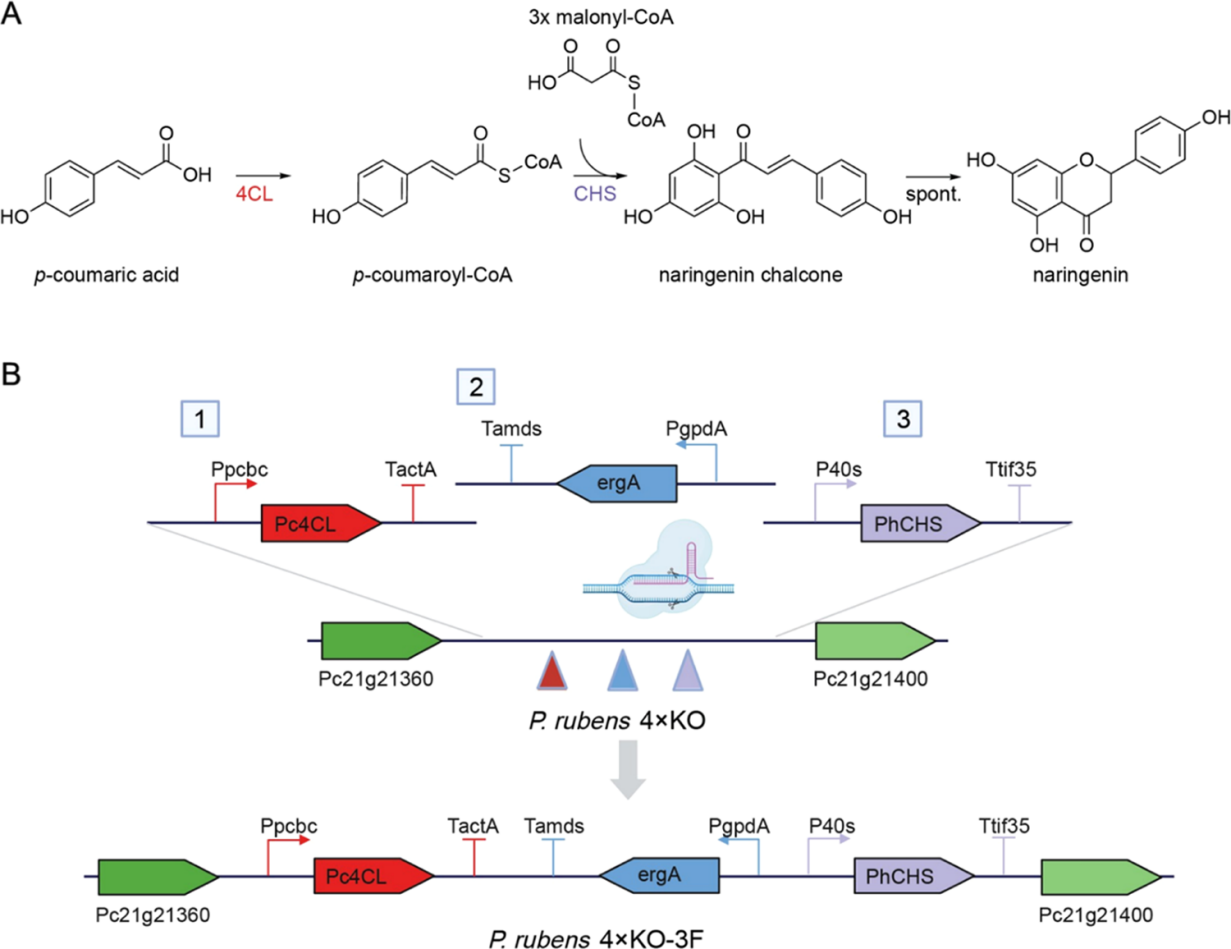
Biosynthesis pathway of naringenin and integration of the naringenin biosynthesis pathway into the *pen* locus of *P. rubens* 4xKO. (A) Biosynthesis pathway of naringenin. (4CL: 4-coumarate: CoA ligase, CHS: chalcone synthase). (B) Scheme for recombination of two (2 and 3) or three (1, 2, and 3) fragments (each fragment possesses homology arms of 100 bp) into the intergenic region between Pc21g21360 and Pc21g21400. The obtained recombination strains were named *P. rubens* 4xKO-2F and *P. rubens* 4xKO-3F.

Therefore, the fermentative production of specific flavonoids, such as naringenin, an important precursor for many flavonoids, has attracted significant attention over the last 15 years. Several studies report the successful production of flavonoids using microbial hosts, among others *Escherichia coli*,^9,10^*Saccharomyces cerevisiae*,^11,12^ and *Yarrowia lipolytica*.^13,14^ Elaborate metabolic engineering campaigns were recently reported to overcome pathway bottlenecks and maximize production yields.^13,15−17^ The major bottleneck in microbial naringenin production appears to be the limitation of free malonyl-Coenzyme A (malonyl-CoA) that is available for secondary metabolism in the host strain.^8^ Malonyl-CoA is the main precursor for fatty acid biosynthesis, an essential process in primary metabolism, and its abundance is strictly regulated to avoid the waste of cellular resources. The key enzyme of naringenin biosynthesis, chalcone synthase (CHS) (Figure 2A), is a type III polyketide synthase and directly competes with fatty acid biosynthesis for malonyl-CoA, since this is one of its natural substrates. Therefore, it has been crucial to increase the malonyl-CoA pool by engineering its upstream pathway and by suppressing fatty acid synthesis in microbial flavonoid producers.^18,19^

The aforementioned bottleneck suggests that using a microbial host that is naturally gifted or already engineered to produce high levels of secondary metabolites relying on malonyl-CoA, could be a good choice to recombinantly produce flavonoids. Therefore, we turned to derivatives of *P. rubens* Wisconsin 54-1255. These derivatives were previously engineered to produce β-lactam antibiotics but were also shown to support the high-level production of cholesterol-lowering statins, which are malonyl-CoA-dependent polyketides.^20,21^ Specifically, we chose the secondary metabolite-deficient derivative of *P. rubens* DS68530, named *P. rubens* 4xKO. This strain was recently constructed via CRISPR/Cas9-based genome editing, via deletion of four highly expressed secondary metabolite gene clusters.^22,23^ The low background of endogenous secondary metabolites simplifies the detection of target molecules, facilitates the downstream purification of the desired product, and prevents valuable resources from being utilized for unwanted secondary metabolites. When characterizing the *P. rubens* 4xKO strain further, Pohl et al. found that it does support the heterologous production of a polyketide product at higher titers than the parental strain, when both were transformed with the same biosynthetic gene cluster.^23^ In these two transformants, they furthermore observed that the gene encoding an ortholog of *S. cerevisiae* acetyl-CoA decarboxylase, gene Pc13g03920, was moderately upregulated in 4xKO compared to the parental strain.^23^ Acetyl-CoA decarboxylase catalyzes the conversion of acetyl-CoA to malonyl-CoA and an increased expression level may increase the availability of malonyl-CoA. Intrigued by these findings, we thought that *P. rubens* 4xKO could be an excellent option for the heterologous production of other polyketides, such as flavonoids. To establish naringenin production in *P. rubens* 4xKO, we integrated the genes encoding CHS and 4-coumarate: CoA ligase (4CL) via CRISPR/Cas9-mediated engineering. After optimizing the media composition and precursor feeding strategy, we achieved high molar yields of naringenin from the fed precursor *p*-coumaric acid. We also observed the ability of *P. rubens* to degrade naringenin and investigated the degradation pathway by metabolomics.^24^

## 2 Materials and Methods

### 2.1 Strains, Media, and Culture Conditions

*E. coli* DH10β strain was used for cloning of transcription units. *P. rubens* 4xKO (Δ*penicillin*-BGC, Δ*chrysogine*-BGC, Δ*roquefortine*-BGC:: *amds*, Δ*hcpA*::*ble*, Δ*hdfA*) strain was used for the heterologous expression of the naringenin biosynthesis genes.^23^ Spores of *P. rubens* stored on lyophilized rice grains were first germinated as precultures in YGG medium (in g/L): KCl, 8.0; glucose, 16.0; yeast nitrogen base, 6.66; citric acid, 1.5; K_2_HPO_4_, 6.0; and yeast extract, 2.0. Secondary metabolite-producing medium (SMP, pH 6.3) was prepared for secondary metabolite production, and the components of the SMP medium are listed below (in g/L): K_2_HPO_4_, 2.12; KH_2_PO_4_, 5.1; CH_3_COONH_4_, 5.0; urea, 4.0; Na_2_SO_4_, 4.0; carbon source, 75.0; and supplemented with 4.0 mL Trace Element Solution,^25^ when appropriate, supplemented with 1.1 μg/mL terbinafine hydrochloride (Sigma-Aldrich) for selection. Protoplasts were recovered after 5 days on selective, solid transformation medium containing (in g/L): sucrose, 375.0; agar, 15.0; glucose, 10.0, and 4.0 mL Trace Element Solution, 27.0 mL Stock Solution A (KCl, 28.8; KH_2_PO_4_, 60.8; NaNO_3_, 240.0; at pH 5.5), 27.0 mL Stock Solution B (MgSO_4_·7H_2_O, 20.8), and pH adjusted around 7.0.^25,26^ For sporulation, purification, or preparation of lyophilized rice batches of *P. rubens* strains, R-agar medium was used, supplemented with 1.1 μg/mL terbinafine hydrochloride, and prepared as following (in g/L): agar 15.0; yeast extract, 5.0; MgSO_4_**·**7H_2_O, 0.05; NaCl, 18.0; CaSO_4_**·**2H_2_O, 0.25; KH_2_PO_4_, 0.06; CuSO_4_**·**5H_2_O, 0.01; NH_4_Fe(SO_4_)_2_**·**12H_2_O, 0.16; added with 7 mL of 85% glycerol and 7.5 mL of sugar beet molasses, provided by DSM-Firmenich (Delft, the Netherlands). All *P. rubens* strains were incubated at 25 °C and 200 rpm in 125 mL baffled flasks (Bellco) for liquid medium.

### 2.2 Plasmid Construction

All plasmids and primers used in this study are summarized in Tables 1 and S1. The genes of Pc4CL, 4-coumarate: CoA ligase from *Petroselinum crispum* (GenBank accession number KX671122.1), and PhCHS, chalcone synthase from *Petunia hybrida* (GenBank accession number KP284563.1) were ordered as synthetic genes from Integrated DNA Technologies (IDT, EU). All vectors were constructed via the Golden Gate technology-based Modular Cloning (MoClo) system using Type IIS restriction enzymes BpiI and BsaI as described previously.^27^ To assemble *Pc4CL* and *PhCHS* into MoClo entry vector pICH41308 (level 0) as pFL_0_1_Pc4CL and pFL_0_2_PhCHS, respectively, both ORF fragments were amplified with KAPA HiFi HotStart ReadyMix (Roche Diagnostics, Switzerland) with primers (Table S1) that carried two BpiI restriction sites at the 5′- and 3′-end. The genes of interest, promoters, and terminators were then constructed into the MoClo transcription unit vectors (level 1), pICH47742 and pICH47761, with the BsaI restriction enzyme (Thermo Fisher Scientific, Waltham, MA).^28^ Both level 0 and level 1 plasmids were constructed to replace the *lacZ* gene of each backbone vector and then transformed into *E. coli* DH10β competent cells. Correctly assembled vectors were identified with blue-white screening, isolated by a miniprep kit (Sigma-Aldrich), and analyzed by sequencing (Macrogen, Europe B.V.).

**Table 1:** Plasmids Used in This Study

plasmid	application	template	origin
pICH41308	level 0 cloning backbone for *pc4cl* and *phchs*		Addgene#47998^27^
pICH47742	level 1 cloning backbone for *pc4cl*		Addgene#48001^27^
pICH47761	level 1 cloning backbone for *phchs*		Addgene#48003^27^
pFTK013	PpcbC Pc21g21380 promoter for *pc4cl*	*P. rubens* DS54468^29^	Addgene#171285^28^
pFTK081	TactA ANIA_06542 *P. rubens* terminator for *pc4cl*	pDSM-JAK-108^30^	Addgene#171353^28^
pFTK012	P40s AN0465 promoter for *phchs*	pDSM-JAK-108^30^	Addgene#171284^28^
pFTK076	Ttif35 Pc22g19890 terminator for *phchs*	pDSM-JAK-108^30^	Addgene#171348^28^
pCP1_45	level 1 vector with *PgpdA-ergA-Tamds* as the terbinafine selection marker	*P. rubens*DS54468^31^	Pohl et al.^23^
pFL_0_1_Pc4CL	level 0 vector with *pc4cl* CDS	pETDuet:: pc4cl	this study
pFL_0_2_PhCHS	level 0 vector with *phchs* CDS	pETDuet:: phchs^32^	this study
pFL_1_1_Pc4CL	level 1 vector with TU for donor DNA		this study
pFL_1_2_PhCHS	level 1 vector with TU for donor DNA		this study
pET28a/Cas9-Cy	Cas9 protein overexpression		Addgene#53261^33^

CDS: coding sequence; TU: transcription unit.

Donor DNA fragments were amplified by PCR reactions from the level 1 plasmids pFL_1_1_Pc4CL, pFL_1_2_PhCHS, and pCP1_45, introducing 100 bp long flanking regions on each end for homologous recombination. All three fragments were integrated into the *P. rubens* 4xKO chromosome at the original penicillin gene cluster chromosomal site (*pen* locus), 5′ of the *penDE* gene Pc21g21370 and 3′ of the *pcbAB* gene Pc21g21390 (Figure 2B).

### 2.3 Fungal Transformations

The preparation of protoplasts, transformation, and colony screening were performed as described previously.^23,26^ Approximately 2 × 10^7^ protoplasts, 8 μg donor DNA, and the preincubated mixture of 27 μg purified Cas9 protein and 4 μL synthesized single-guide RNAs (sgRNAs) were mixed for transformation. Cas9 protein was overexpressed in *E. coli* T7 Express lysY (New England Biolabs, U.K.) from pET28a/Cas9-Cys (Addgene plasmid # 53261^33^), and purified via Ni-NTA affinity chromatography. The T7-sgRNA DNA templates were generated by PCR amplification with a pair of overlapping primers, and then the MEGAscript T7 Transcription Kit (Thermo Fisher Scientific) was used to synthesize sgRNA. The transformed protoplasts were plated onto transformation media with terbinafine and incubated for 5–6 days at 25 °C with increased humidity for recovery.^23,26^ Transformants were screened via colony PCR (Table S1, Figure S1) using Phire Green HotStart II PCR Master Mix (Thermo Fisher Scientific) to confirm the integration of donor DNA elements at the desired genomic locus and verified by sequencing. Correct transformants were grown on terbinafine-containing R-agar plates for 3–5 days for sporulation and purified by several rounds of sporulation and genotype confirmation, to obtain genetically pure clones. For long-term storage, spores were inoculated on sterile long-grain rice (Ben’s Original), lyophilized, and stored at room temperature.^23,25^

### 2.4 Fermentation Conditions, Secondary Metabolite Extraction and Analysis, and Biomass Measurement

The fermentation was performed in a liquid culture. Three grains of rice with immobilized fungal spores (1.7 × 10^7^ spores/grain) were inoculated for 24 h in 3 mL of YGG medium before being transferred into 22 mL of SMP medium in a 125 mL flask. After 1–4 days of cultivation, the precursor *p*-coumaric acid was added to the culture, and samples were taken periodically over the course of several days as described in the Results section. To quantify the naringenin concentration, 1 mL of culture was taken from the flask and mixed with 1 mL of methanol solution (100% methanol with 0.1% TFA). The mixture was centrifuged for 10 min at 10,000*g*, and the supernatant was used for HPLC analysis. To characterize secondary metabolites of *P. rubens* 4xKO variants, 2 mL samples were taken from the flask and extracted twice with 4 mL of ethyl acetate. The organic phase was collected and evaporated. After evaporation, 1 mL of 50% methanol (in water) was added to dissolve the crude extract and filtered by a 0.45 μm PTFE syringe filter to remove insoluble particles prior to analysis. The samples were stored at −20 °C if not used immediately for high-performance liquid chromatography-coupled mass spectrometry (HPLC-MS) analysis. For the biomass determination, mycelia were harvested by vacuum filtration over 0.45 μm cellulose filters (Sartorius, Germany) at the indicated times. The collected biomass was dried at 60 °C for 60 h and then weighed.

### 2.5 Analysis and Quantification of Target Compounds

Chemical standards for *p*-coumaric acid, naringenin, and phloretin were purchased from Sigma-Aldrich. Quantification of naringenin from the fermentation broths was achieved by high-performance liquid chromatography (HPLC, Shimadzu LC-10AT, equipped with an SPD-20A photodiode array detector) using a previously reported HPLC method.^32^ Briefly, 10 μL of samples were injected into an Agilent Eclipse XDB-C18 (5 μm, 4.6 × 150 mm) column and separated with the following mobile phases: A: water +0.1% trifluoroacetic acid (TFA); B: acetonitrile +0.1% TFA. The following gradient was used: 15% B for 3 min, 15- 90% B over 6 min; 90% B for 2 min; 90–15% B over 3 min, 15% B for 4 min; flow rate: 1 mL/min. Phloretin and naringenin were identified by comparison with chemical standards. The peak areas were integrated and converted to concentrations based on calibration curves obtained with chemical standards (Figure S2).

The identity of secondary metabolites was assessed by utilizing liquid chromatography–mass spectrometry (LC-MS) with a Waters Acquity Arc HPLC-MS system equipped with a 2998 PDA detector and a QDa single-quadrupole mass detector. Samples (1 μL) were injected into and separated over an Xbridge BEH C18 (3.5 μm, 2.1 × 50 mm) column with the following mobile phases: A: water +0.1% formic acid (FA); B, acetonitrile +0.1% FA. The following gradient was used: 5% B for 2 min, 5–50% B over 15 min, 50–90% B over 4 min; 90% B for 3 min, 90–5% B over 6 min; flow rate: 0.25 mL/min. MS analysis was carried out in positive mode, with the following parameters: probe temperature of 600 °C; capillary voltage of 1.0 kV; cone voltage of 15 V; scan range 100–1250 *m*/*z*.

High-resolution tandem MS analyses were performed with a Shimadzu Nexera X2 HPLC system with binary LC20ADXR interfaced to a Q Exactive Plus Hybrid Quadrupole-Orbitrap Mass Spectrometer (Thermo Scientific). A 100 × 2.1 mm Kinetex EVO C18 reversed-phase column with 2.6 μm 100 Å particles (Phenomenex) was used for separation. The column and autosampler temperatures were set at 50 and 10 °C, respectively. The injection volume was 2 μL, and the flow rate was set at 0.25 mL/min. Mobile phases were used as same as HPLC-MS. The following gradient was used: 5% B for 2 min, 5–50% B over 32 min, 50–90% B over 8 min; 90% B for 3 min, 90–5% B over 5 min. MS and MS/MS analyses were performed with electrospray ionization in positive mode at a spray voltage of 3.5 kV, a sheath gas pressure of 60 psi, and an auxiliary gas flow of 11 arbitrary units. The ion transfer tube temperature was 300 °C. Spectra were acquired in data-dependent mode with a survey scan at *m*/*z* 100–1650 at a resolution of 70,000 followed by MS/MS fragmentation of the top 5 precursor ions at a resolution of 17,500. A normalized collision energy (NCE) of 30 was used for fragmentation, and fragmented precursor ions were dynamically excluded for 10 s.

## 3 Results

### 3.1 Validation of Naringenin Production in Engineered *Penicillium rubens* 4xKO-2F and -3F

It is well established that the expression of a chalcone synthase (CHS) and a 4-coumarate: CoA ligase (4CL) in heterologous hosts in bacteria and yeast is sufficient to support the production of naringenin from fed *p*-coumaric acid. In particular, PhCHS from *P. hybrida* and Pc4CL from *P. crispum* are reported to be a highly efficient combination of enzymes.^10,34,35^ Since *P. rubens* has been reported to express several native CoA ligases, with one of them even accepting *p*-coumaric acid as a substrate *in vitro*,^36^ we first tested if both plant enzymes need to be expressed in this new host to produce naringenin. We constructed the two strains *P. rubens* 4xKO-2F and −3F by genomic integration of the PhCHS encoding gene or the PhCHS and Pc4CL encoding genes, respectively, into the *pen* locus (Figure 2B) and confirmed the integration by colony PCR (Figure S1).

**Figure 3:**
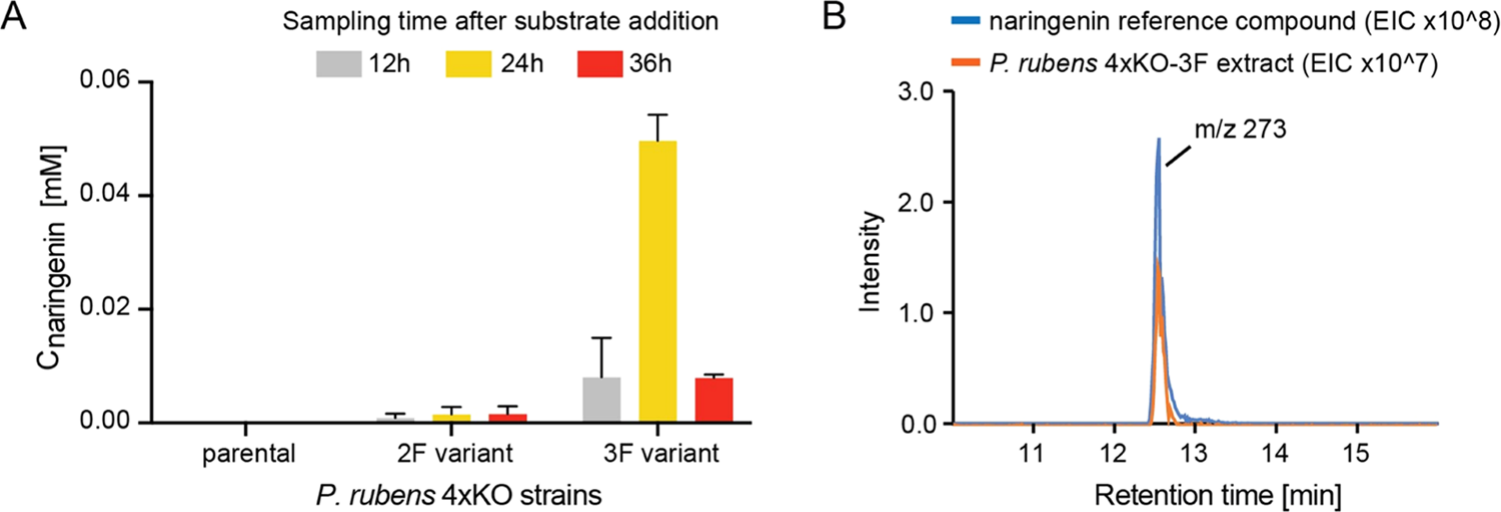
Comparison of *P. rubens* 4xKO variants toward naringenin production and chromatography of naringenin standard and samples. (A) Naringenin titers detected in culture extracts of *P. rubens* 4xKO strains taken at different time points after precursor feeding. Data points represent mean ± SD of biological replicates, *n* = 3. (B) Extracted ion chromatograms of the culture extract of *P. rubens* 4xKO-3F (orange) and the naringenin reference compound (blue) ([M + H]^+^ at *m*/*z* 273, RT = 12.57 min).

We then performed fermentations with the standard laboratory protocol in selective SMP medium with 75 g/L lactose as the carbon source, pH 6.3, and with 1 mM *p*-coumaric acid as the precursor for naringenin production. When analyzing the culture extracts of the engineered variants by HPLC-MS, we observed a peak with the same retention time (RT) and mass-to-charge ratio (*m*/*z*) as those of the naringenin standard (Figure 3). However, the titers in the *P. rubens* 4xKO-2F culture were extremely low. This suggests that one of the native CoA ligases can support the conversion of fed *p*-coumaric acid to coumaroyl-CoA, yet not efficiently. Coexpression of the two plant enzymes appears to be the better strategy to produce naringenin in *P. rubens*. In this coexpression strain (−3F), the highest titer of naringenin was about 0.05 mM 24 h after precursor addition. From 24 to 36 h, the naringenin concentration decreased. This indicates that naringenin might also be rapidly degraded by the growing fungal cultures.

### 3.2 Optimization of Precursor Feeding Time

**Figure 4:**
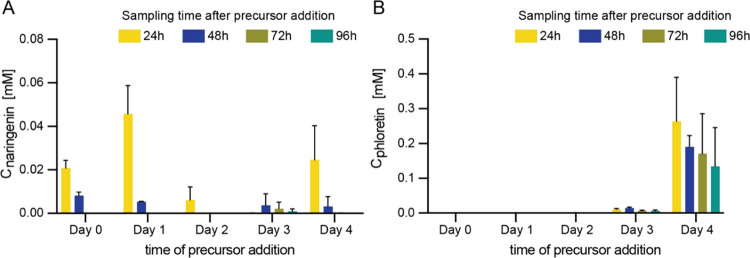
Time course of naringenin and phloretin accumulation in *P. rubens* 4xKO-3F with different precursor feeding strategies. Fermentations were performed in SMP medium (lactose as a carbon source, pH 6.3) with 1 mM *p*-coumaric acid fed after 0, 1, 2, 3, and 4 days of cultivation. Samples were taken every 24 h after feeding precursor, then extracted and analyzed by HPLC-MS. Data points represent mean ± SD of biological triplicates, *n* = 3. (A) Naringenin titers (target compound). (B) Phloretin titers (side product).

Based on the results shown above, we assumed that *P. rubens* degraded naringenin from 24 to 36 h after precursor addition, when the fungal biomass was still increasing (data not shown). Thus, we set out to explore how the precursor feeding time influences naringenin production. We varied the precursor feeding time point to 0, 1, 2, 3, or 4 days after inoculation of the precultures in the selective SMP medium, while keeping the other fermentation parameters the same. We took samples of the cultures every 24 h for several days and analyzed the extracts by LC-MS. The results show that the highest naringenin titer was obtained in the cultures when the precursor was fed after 1 day of cultivation at the 24 h sampling time point (Figure 4A, around 0.05 mM naringenin). Surprisingly, we also detected another dominant compound in the cultures that were fed with the precursor after three and four days of cultivation (Figure 4B). Based on the *m*/*z* ratio of 275.0929 ([M + H]^+^) in tandem high-resolution MS (Figure S3), we predicted an elemental composition of C_15_H_14_O_5_ and hypothesized that this compound is phloretin, a reduced derivative of naringenin chalcone. We confirmed this hypothesis by comparing the retention time and *m*/*z* value to those of the commercially available reference compound. Upon closer inspection of all sample chromatograms, we also noticed that no phloretin can be detected in the samples under the other feeding conditions. One possible explanation for this phenomenon could be that in the absence of a chalcone isomerase, the naringenin chalcone generated by *P. rubens* 4xKO-3F cannot be converted into naringenin immediately and is then reduced to phloretin by native reductases. Thus, we concluded that feeding of *p*-coumaric acid after 24 h of cultivation in SMP medium followed by harvesting the culture the next day was the best strategy.

### 3.3 Optimization of Naringenin Production

**Figure 5:**
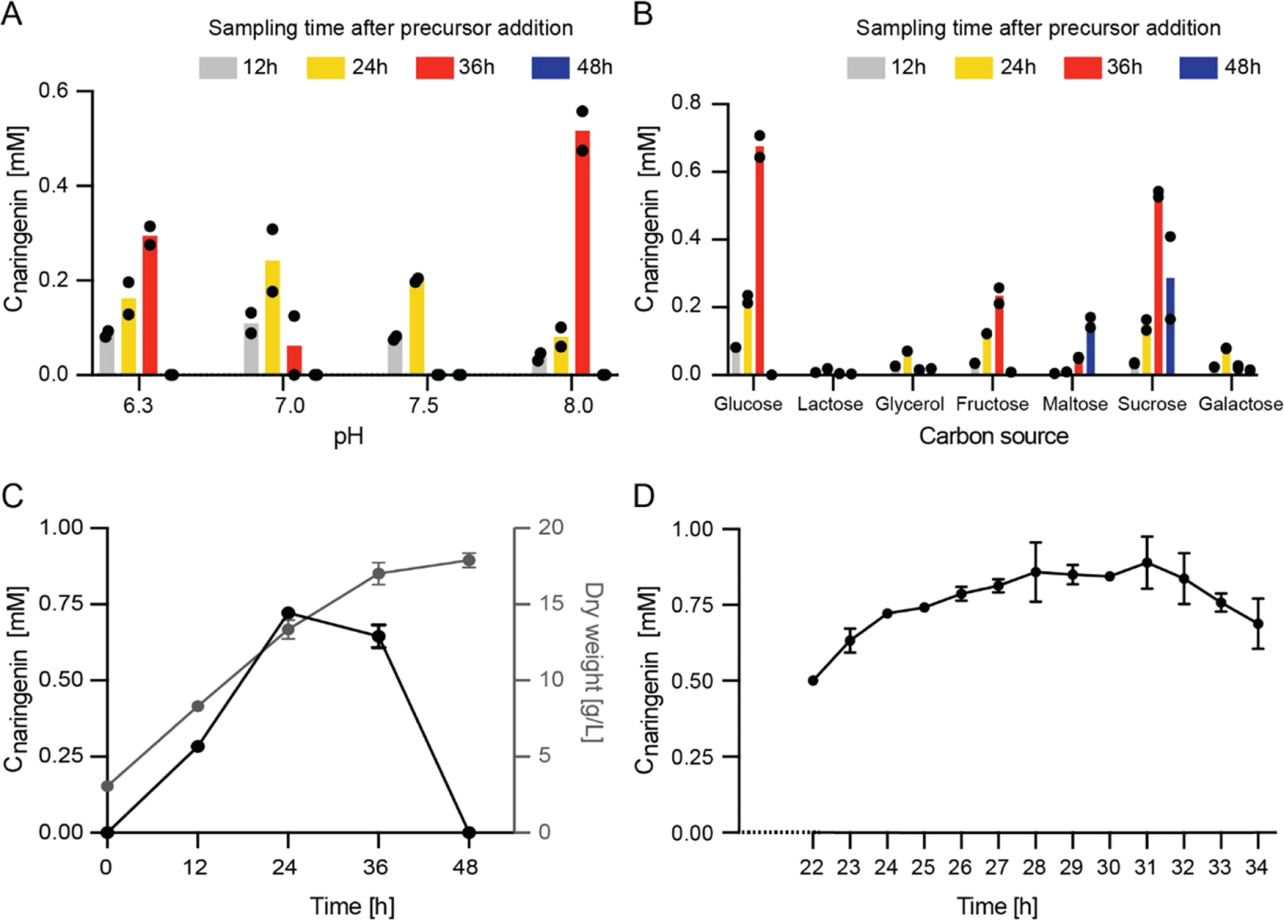
Time course of naringenin accumulation in *P. rubens* 4xKO-3F in different media. 1 mM *p*-coumaric acid was fed after 24 h of cultivation. (A) Naringenin titers obtained from cultures with different medium pH and glucose as a carbon source (*n* = 2). (B) Naringenin titers obtained from cultures with different carbon sources and a medium pH of 8.0 (*n* = 2). (C) Naringenin titers obtained from cultures in optimized media (black) and *P. rubens* 4xKO-3F biomass accumulation during naringenin production sampled every 12 h (gray) (*n* = 3). (D) Naringenin titers obtained from cultures in optimized media sampled every hour from 22 to 34 h after precursor addition (*n* = 3). The highest titer of naringenin reached 0.88 mM from 1 mM *p*-coumaric acid.

Since the maximum titers of naringenin were very low with only 5% of the fed *p*-coumaric acid converted into the target product, we then set out to further improve the fermentation conditions. It is generally accepted that the media composition and pH can change the secondary metabolite profiles in filamentous fungi, and we therefore set out to explore different growth media.^37,38^ In a quick prescreening of carbon sources, we saw that utilizing glucose rather than lactose notably increased the final titers of naringenin (data not shown). So, for the pH optimization, the fermentations were carried out in SMP media with glucose as a carbon source and the pH of the phosphate buffer ranged from 6.3 to 8.0. We fed 1 mM *p*-coumaric acid after 24 h of cultivation in the modified SMP medium and took samples of the cultures every 12 h for HPLC analysis. The highest titers achieved with the different media were 0.3 mM (pH 6.3, 36 h after precursor feeding), 0.3 mM (pH 7.0, 24 h after precursor feeding), 0.2 mM (pH 7.5, 36 h after precursor feeding), and 0.55 mM (pH 8.0, 36 h after precursor feeding) (Figure 5A), indicating that pH 8.0 gives the best result. Two days after precursor feeding, no naringenin was detected in any of the cultures, again demonstrating the subsequent degradation of the product.

After confirming the best pH condition, we repeated the screening for the best carbon source of the fermentation media and examined naringenin production under fermentation with different carbon sources (glucose, lactose, glycerol, fructose, maltose, sucrose, and galactose) in the selective SMP medium (pH 8.0) (Figure 5B). Consistent with our previous result, the naringenin titer in lactose-containing medium was lower, while glucose-containing medium provided the highest titer (0.75 mM). Cultivation in fructose- or sucrose-containing media also supported higher conversion of fed *p*-coumaric acid and accumulation of naringenin than lactose. Thus, changing the carbon source and the pH of the media gave a boost in naringenin titer at the 36 h time point by 1 order of magnitude. From 36 to 48 h, the concentration of naringenin in the cultures decreased.

To further improve the molar yield of naringenin from 1 mM *p*-coumaric acid, we next performed another time course experiment to optimize the time point for harvesting the culture and characterize the growth phenotype of the *P. rubens* 4xKO-3F strain. Since the antifungal terbinafine was still used in all fermentations described thus far, we noticed poor growth in most experiments and decided to proceed without this additive. The genes encoding PhCHS and Pc4CL are integrated into the genome, and the resulting strain was thoroughly purified. Therefore, this additional selection is not necessary. We cultivated the engineered strain under the optimized conditions (modified SMP medium with pH 8.0 and glucose as carbon source), fed the precursor 1 mM *p*-coumaric acid after 24 h, and took samples at a 12 h interval, with additional sampling every hour between 22 and 34 h (Figure 5C,D). In the first 24 h, naringenin accumulated in the fermentation broth (from 0 to 0.7 mM), and the biomass increased linearly from 3.0 to 13.3 g/L. Then from 24 to 31 h, the titer of naringenin reached a peak (around 0.88 mM naringenin), and the biomass accumulation slowed down (from 13.3 to 17.0 g/L in 12 h). After 31 h of cultivation, the concentration of naringenin began to decrease, and the culture reached the stationary phase. At the 48 h time point, no naringenin was detected in the culture. The highest titer and molar yield (0.88 mM and 88%, respectively) were much higher than in our previous experiments and indicate that *P. rubens* can be pushed to produce high amounts of naringenin with an almost stoichiometric conversion of *p*-coumaric acid into naringenin.

To further rationalize the positive effect of the new media composition, we analyzed naringenin titers, biomass accumulation, and the pH of the cultures in a more detailed time course experiment in direct comparison with the original media composition (Figure S4). We observed that both media yield approximately the same cell dry weight in the stationary phase of the fermentation, although the growth curve in the original media (lactose, pH 6.3) shows a biphasic shape with unexpectedly low cell dry weight between 24 and 48 h. This “kink” in the growth curve coincides with an increase in the pH of the culture by 1.5 units. Thus, we conclude that the improved naringenin titers upon media optimization are not related to an overall boost in the growth of *P. rubens* 4xKO-3F, however, the differences in the growth kinetics in the main naringenin production phase (0–36 h) may be beneficial.

Next, we investigated whether feeding with higher concentrations of the *p*-coumaric acid precursor would further increase the naringenin titers. We fed 1, 2.5, 5, and 7.5 mM *p*-coumaric acid in the optimized media and analyzed naringenin titers, biomass formation, and the pH of the cultures over time (Figure S5). Overall, we observed similar growth curves and pH traces for all cultures with maybe a slightly lower cell dry weight at the 24 h time point in the presence of the higher precursor concentrations. In all cultures, the highest naringenin titers were measured at 36 h, with the highest molar yields achieved for the 1 mM *p*-coumaric acid condition. The overall highest titer was measured for the 2.5 mM condition (1.5 mM naringenin), while higher precursor loads led to lower naringenin titers (1.32 and 1 mM, respectively).

### 3.4 Naringenin Degradation in *P. rubens* 4xKO

Since fungi play an important role in natural ecosystems for degrading biomass, it is well known that they have catabolic pathways to degrade and utilize plant polymers and small organic compounds. In the literature, several filamentous fungi and yeasts have been described to modify flavonoids by oxidative processes or glycosylation,^39,40^ yet complete degradation of the scaffold has only been reported in bacteria.^41,42^ For example, Marin et al. investigated naringenin degradation in the β-proteobacterium *Herbaspirillum seropedicae* SmR1.^41,42^ They pinpointed a gene cluster responsible for the degradation and identified key intermediates by high-resolution tandem MS and nuclear magnetic resonance, while Braune et al.^43^ demonstrated that an oxygen-sensitive NADH-dependent reductase from *Eubacterium ramulus* could cleave naringenin, eriodictyol, liquiritigenin, and homoeriodictyol.

**Figure 6:**
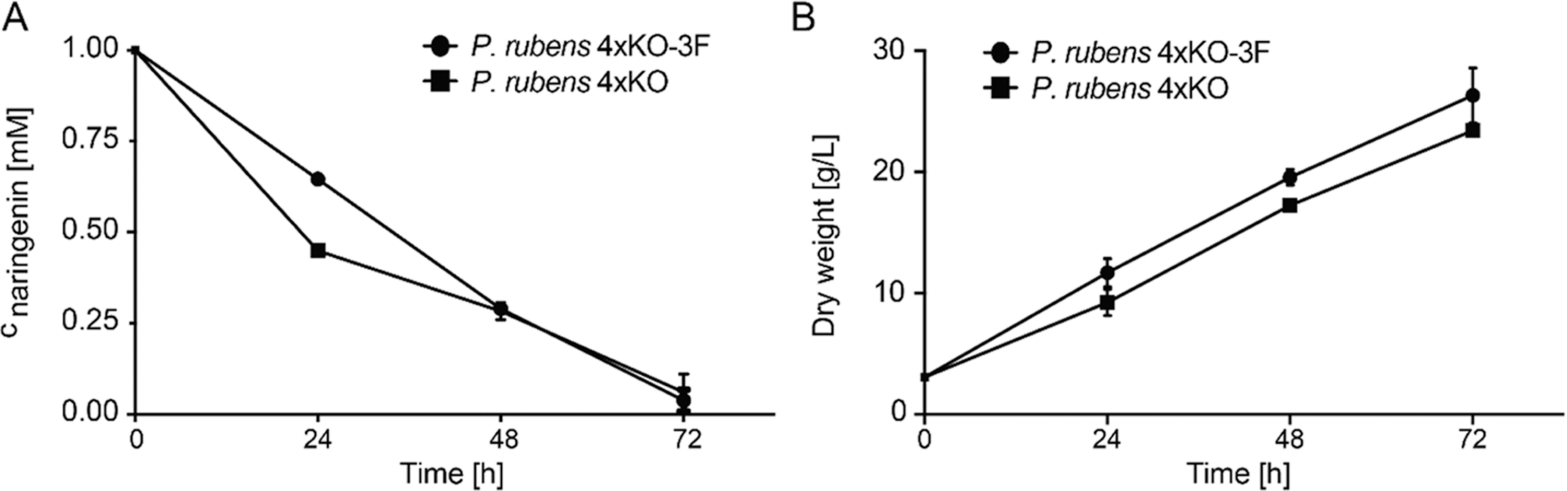
Time course of naringenin degradation in *P. rubens* 4xKO and *P. rubens* 4xKO-3F. Naringenin (1 mM) was fed after 24 h of cultivation in optimized SMP media. Data points represent mean ± SD of biological replicates, *n* = 3. (A) Naringenin titer over time. (B) Biomass accumulation over time.

With these studies in mind, we performed a time course experiment to investigate whether any of the heterologously expressed plant enzymes were involved in naringenin degradation and to investigate the intermediates of naringenin degradation. We cultured *P. rubens* 4xKO and *P. rubens* 4xKO-3F for 1 day in modified SMP medium as described above (pH 8.0, glucose as carbon source) before adding 1 mM naringenin. Next, samples were collected every 24 h for HPLC and HPLC-MS analyses, with an additional sample taken after 6 h. For both strains, we observed that the concentration of naringenin decreases linearly over 72 h until most naringenin was degraded (final concentration 0.06 mM in cultures of the parental strain and 0.037 mM in the 3F strain) (Figure 6A). Furthermore, the biomass increases at a steady rate even after 48 h to a higher amount of cell dry weight than in all previous experiments (23.5 g/L for parental strain and 26 g/L for 3F) (Figure 6B). Since the parental strain degrades naringenin, it can be concluded that the heterologously expressed plant enzymes are not required to degrade naringenin.

**Figure 7:**
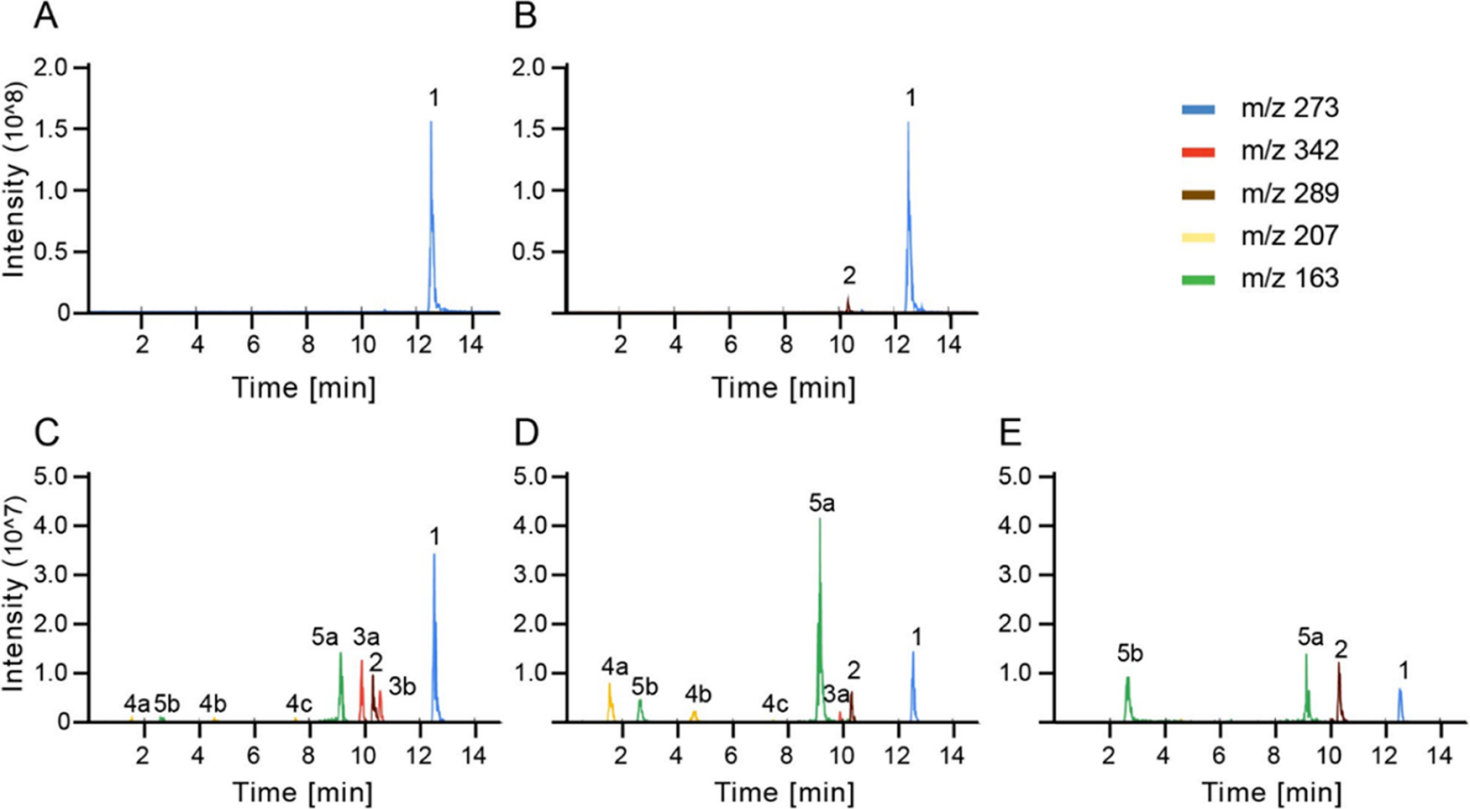
Extracted ion chromatograms of the major intermediates from naringenin degradation by *P. rubens* 4xKO analyzed by HPLC-MS: (A) 0 h, (B) 6 h, (C) 24 h, (D) 48 h, (E) 72 h time point. The strain was grown at 25 °C in modified SMP medium containing 1 mM naringenin.

**Figure 8:**
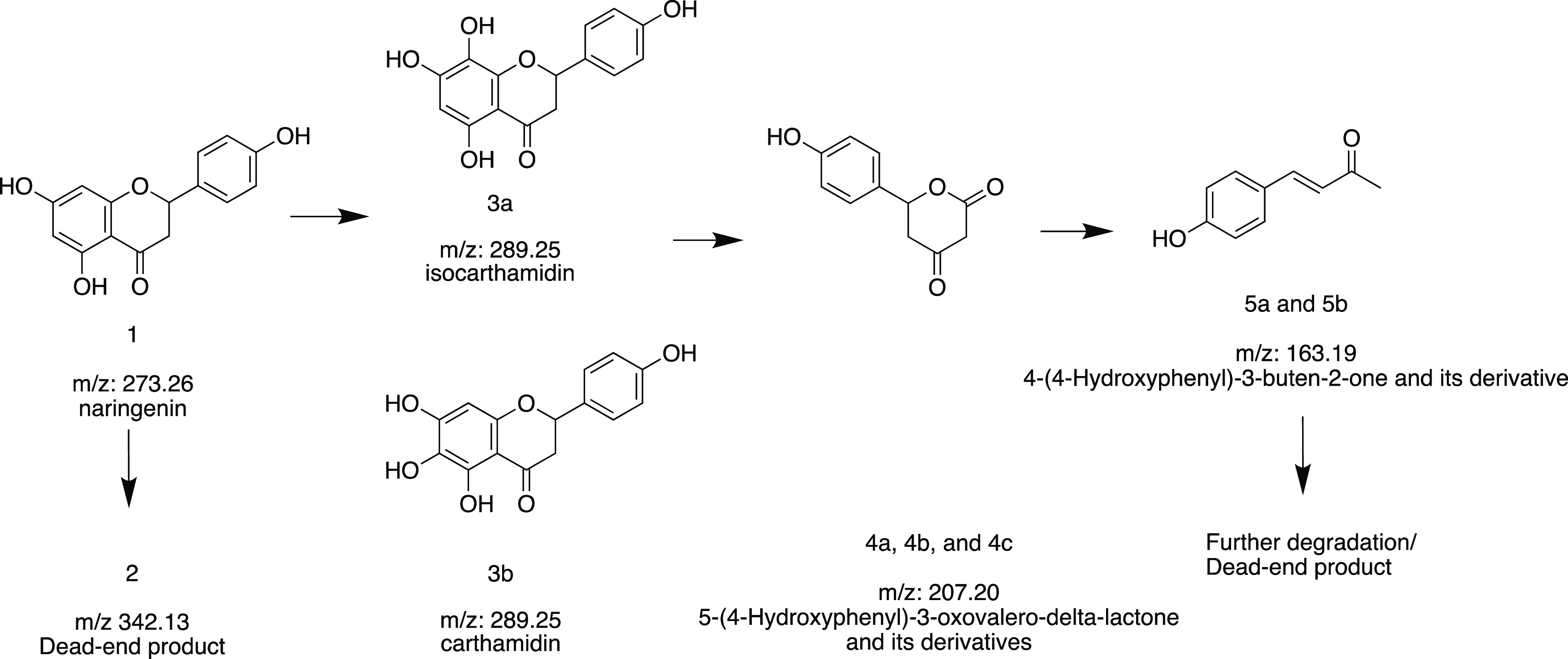
Proposed naringenin degradation pathway in *P. rubens* 4xKO.

Next, we compared the peak areas, retention times, and *m*/*z* values of new peaks in the low-resolution HPLC-MS chromatograms of the various samples from both strains and found that there were also no variations in the degradation products of naringenin between *P. rubens* 4xKO and *P. rubens* 4xKO-3F. At time point 0 h, we only detected naringenin in the culture (compound 1), but after 6 h of cultivation, we observed compound 2 at RT 10.30 min (*m*/*z* 342 ([M + H]^+^)) (Figure 7A,B). Compound 2 persisted in the culture media for 72 h, suggesting that it could be a dead-end product in *P. rubens* 4xKO. After 24 h of cultivation, compounds 3a/b (*m*/*z* 289), 4a–c (*m*/*z* 207), and 5a/b (*m*/*z* 163) were found in the culture (Figure 7C). After 72 h, compounds 4a-c were degraded, but compounds 2, 5a, and 5b remained in the culture media (Figure 7D). Already based on the low-resolution data, we noticed that compounds 2, 4a–c, and 5a/b do not correspond with the flavonoid derivatives previously described for fungal cultures.^40^ Thus, to further characterize the major degradation intermediates, we performed high-resolution tandem MS on the samples and compared the data to natural product databases and literature reports of flavonoid degradation in microbes^40^ (Table 2). Even with this more detailed analysis, we did not find a match in the literature for compound 2. One of the highest-ranked predicted molecular formulas, C_19_H_19_NO_5_, and the MS2 fragmentation pattern may suggest that it is a nitrogen-containing derivative of naringenin with a substitution on the A-ring. We were, however, unable to find further information on such a modification in nature and therefore refrain from further speculation. The analysis of compounds 3a/b showed that they have the same *m*/*z* ratio within precision of the instrument (289.0728 ([M + H]^+^)) and thus have the same predicted molecular formula (C_15_H_12_O_6_). Based on the fragmentation pattern in MS2 with two characteristic fragments (*m*/*z* 169.0147 and 147.0453; Figure S6), we hypothesize that these two features likely correspond to isocarthamidin and carthamidin with an additional hydroxyl group on the A-ring compared to naringenin.^44−46^ Compounds 4a–c have identical *m*/*z* of 207.0669 ([M + H]^+^) and thus the same predicted molecular formula (C_11_H_10_O_4_). Since the three features also share the most common fragment ions, we hypothesize that they are structurally related compounds that already fragment in MS1 to form the common 207.0669 parent ion. Similarly, compounds 5a/b have an identical *m*/*z* of 163.0768 ([M + H]^+^) with the same predicted molecular formula (C_10_H_10_O_2_) and they share the most abundant fragment ions. Based on the *m*/*z* and comparison to the literature, we hypothesize that compounds 3a/b, 4a–c, and 5a/b are structurally related to the intermediates that were described for the degradation of naringenin in *H. seropedicae* SmR1.^42^ In *H. seropedicae* SmR1, naringenin degradation begins with hydroxylation of the A-ring to form (iso)carthamidin, followed by further hydroxylation on C8, and the loss of the A-ring putatively as oxaloacetate to form the intermediate 5-(4-Hydroxyphenyl)-3-oxovalero-delta-lactone. This lactone is then hydroxylated in the former C2 position of the former B–C ring, the C-ring opens, is decarboxylated, and dehydrated to form the final product 4-(4-Hydroxyphenyl)-3-buten-2-one.^42^ In the cultures of *P. rubens* 4xKO, we detected the expected *m*/*z* values for the hydroxylated flavonoid (*m*/*z* 289.0728 [M + H]^+^, compounds 3a/b), the lactone intermediate (*m*/*z* 207.0503 [M + H]^+^, compounds 4a-c) and the final product (*m*/*z* 163.0768 [M + H]^+^, compounds 5a/b) but not for the other intermediates. This may suggest that flavonoid degradation in *P. rubens* 4xKO follows a similar pathway as described for *H. seropedicae* SmR1 (Figure 8). The oxaloacetate that is released in this proposed pathway may feed into primary metabolism and thus boost biomass formation.

**Table 2:** Characteristics of the Nine Major Peaks Detected via HPLC-MS and High-Resolution Tandem MS in the Extracts of *P. rubens* 4xKO Cultured in the Presence of 1 mM Naringenin

				MS/MS fragment ions		
peak/compound no.	RT^a^ [min]	molecular formula	[M + H]^+^ (error [ppm])	[M + H]^+^ [*m*/*z*]	Intensity, %^b^	best match based on literature
1	12.55	C_15_H_12_O_5_	273.0776 (4.77)	273.0781	100	naringenin
				153.0196	55	
				147.0453	40	
2	10.30	C_19_H_19_NO_5_	342.1364 (6.58)	205.0513	100	unknown
				342.1364	50	
				325.1096	50	
				222.0782	30	
				147.0453	15	
3a	9.89	C_15_H_12_O_6_	289.0728 (5.49)	289.0730	100	isocarthamidin
				169.0147	60	
				147.0453	30	
3b	10.55	C_15_H_12_O_6_	289.0728 (5.49)	289.0730	100	carthamidin
				169.0147	60	
				147.0453	30	
4a	1.61	C_11_H_10_O_4_	207.0669 (5.63)	147.0454	100	5-(4-hydroxyphenyl)-3-oxovalero-delta-lactone or its derivatives^c^
				184.9710	60	
4b	4.56	C_11_H_10_O_4_	207.0669 (5.63)	147.0454	100	
				184.9710	90	
				171.0455	30	
				208.9455	20	
4c	7.47	C_11_H_10_O_4_	207.0669 (5.63)	147.0453	100	
				184.9709	80	
5a	2.62	C_10_H_10_O_2_	163.0768 (5.49)	163.0768	100	4-(4-hydroxyphenyl)-3-buten-2-one or its derivatives^c^
				145.0661	40	
5b	9.12	C_10_H_10_O_2_	163.0768 (5.49)	163.0768	100	
				145.0661	40	

a Retention times as shown in Figure 7 with HPLC-MS data.

b Peak height relative to peak height of main fragment.

c Compound names and structures are based on *m*/*z* values and fragment ion matches with the compounds proposed to be part of the naringenin degradation pathway in ref (42)(42).

## 4 Discussion

In this study, we set out to assess the potential of *P. rubens* derivative strains to produce the polyketide naringenin, which is an important intermediate in the flavonoid metabolic network and a natural product with various health benefits.^2^ By integrating only two plant genes encoding for two flavonoid pathway enzymes into the *P. rubens* 4xKO genome, we were able to detect a low level of naringenin produced from a fed precursor. After optimization of the fermentation conditions (precursor feeding time, medium pH, and carbon source), we achieved a titer of 0.88 mM naringenin, which corresponds to an 88% molar yield from the fed precursor *p*-coumaric acid in flask fermentation. We observed that changing the carbon source from lactose, which is optimal for penicillin production, to glucose led to an increase in naringenin titer by 1 order of magnitude. Changing the pH of the medium from 6.3 to 8.0 led to a further increase by about 1.5-fold. The media composition, especially the carbon source, affects the growth kinetics of *P. rubens*, and since the heterologous pathway genes in the 3F strain are constitutively expressed, this may have a direct impact on the availability of the biocatalysts. It is also possible that the modified medium slows down competing pathways, for example by negatively affecting the expression of native monooxygenases and reductases that facilitate the degradation of the newly built product.

In fact, we observed the rapid degradation of the newly formed naringenin throughout our experiments. We confirmed that the parental strain is capable of degrading fed naringenin, which suggests that the heterologously expressed plant enzymes do not contribute to the degradation. When analyzing the intermediates of naringenin degradation, we observed that some of them differ from known intermediates from fungal conversions of flavonoids reported before.^40^ Instead, they are similar to the degradation products described for *H. seropedicae* SmR1.^42^ In this β-proteobacterium, it was demonstrated that a FAD-dependent monooxygenase, FdeE (Hsero_1007), performs the first catalytic step forming the 8-hydroxylated intermediate, and that a dioxygenase, FdeC (Hsero_1005), is involved in cleaving the A-ring.^41,42^ Several other enzymes encoded in the same gene cluster were further implicated in the degradation pathway, yet no experimental verification has been reported.^42^ Although we were unable to identify a syntenic gene cluster in *P. rubens* Wisconsin 54-1255 genome (GCA_000226395),^47^ we found several monooxygenases and cupin-domain enzymes that could catalyze these reactions in this degradation pathway. It is, of course, also possible that filamentous fungi use different enzymes to achieve the same result. Therefore, a deeper investigation of the homologues of the individual enzymes is necessary to pinpoint the responsible genes in *P. rubens*. Once these are identified, it may be possible to eliminate this competing pathway and, thereby, engineer *P. rubens* into an even more powerful cell factory for flavonoids. Even in this unoptimized *P. rubens* strain, we have achieved final titers (0.88 mM, or 239 mg/L) and molar yields (88%) for naringenin that are competitive compared to previous reports in shake flask experiments with other microbial hosts.^8^ In *E. coli*, Xu et al. reported a naringenin titer of 474 mg/L (about 1.74 mM naringenin) from 2.6 mM *p*-coumaric acid after improving the intracellular availability of malonyl-CoA.^18^ Leonard et al. reported a naringenin titer of 155 mg/mL (0.55 mM) from 3 mM *p*-coumaric acid when employing an alternative carbon assimilation pathway and inhibiting competitive pathways to increase precursor and cofactor supply.^48^ In *S. cerevisiae*, Gao et al. produced 1.2 g/L (approximately 4.44 mM) naringenin from 15.24 mM *p*-coumaric acid by altering the promoters of the biosynthetic enzymes.^15^ Under fed-batch conditions, Mao et al. achieved 2.05 g/L naringenin produced without precursor addition, when employing an optimized strain with a dual dynamic control system to autonomously regulate the synthesis of *p*-coumaric acid and the supply of malonyl-CoA.^16^ In *Y. lipolytica,* Akram et al. obtained 0.63 mM naringenin from 1.22 mM *p*-coumaric acid, which was a 52% molar yield in flask fermentation, whereas only a 12% conversion was obtained in test tube fermentation.^35^ Palmer et al. designed a *de novo* naringenin-producing *Y. lipolytica* strain which can produce 3.3 mM of naringenin in a 3 L batch bioreactor.^14^ These remarkable efforts in recent years show that our *P. rubens* 4xKO-3F strain is an interesting starting point that performs on par with other microbial hosts in shake flask conditions. Using a controlled bioreactor environment as well as silencing the competing degradation pathway will make our *P. rubens* 4xKO-3F an even more competitive microbial cell factory for the production of flavonoids.

In our initial experiments before optimizing the timing of precursor feeding and media composition, we also observed phloretin as a byproduct. Phloretin is a chemical widely applied in medical and cosmetic industries due to its skin-lightening property.^49,50^ The concentration of phloretin reached approximately 0.25 mM from 1 mM *p*-coumaric acid, highlighting again the potential of the engineered *P. rubens* 4xKO-3F for the production of chalcones and flavonoids without further manipulation of the intrinsic malonyl-CoA pool. By integration of additional genes encoding upstream or downstream enzymes, it will be possible to extend this minimal pathway. For instance, integrating a gene for tyrosine ammonia lyase would allow *de novo* synthesis of naringenin from *P. rubens* primary metabolites. Expanding the pathway with flavone synthase would give access to flavones from naringenin, while other oxidoreductases would lead to other types of flavonoids such as flavonols or anthocyanins.^8^

## Supporting Information Available

Gene and primer sequences, evidence for genomic integration, HPLC calibration curves and high-resolution tandem MS analysis for phloretin, isocarthamidin, and carthamidin, time course of naringenin accumulation, dry weight, and medium pH of *P. rubens* 4xKO-3F in two different versions of media (lactose, pH 6.3 versus glucose pH 8.0), time course of naringenin accumulation, dry weight, and medium pH of *P. rubens* 4xKO-3F fed with different concentrations of *p*-coumaric acid in SMP media (glucose, pH 8.0) (PDF)

## Abbreviations and Nomenclature

4CL: 4-coumarate: CoA ligase
CHS: chalcone synthase
CRISPR: Clustered Regularly Interspaced Short Palindromic Repeats
EIC: Extracted ion chromatogram
HR: homologous recombination
HPLC: High-Performance Liquid Chromatography
*m*/*z*: mass-to-charge ratio
MS: mass spectrometry
RT: retention time
TFA: trifluoroacetic acid
