# Supplementary material for: Heterologous Naringenin Production in the Filamentous Fungus Penicillium rubens
Source: J Agric Food Chem. 2023 Dec 16;71(51):20782–92. doi: 10.1021/acs.jafc.3c06755 (PMC10755750; doi:10.1021/acs.jafc.3c06755)
Supplement: Supplementary file 1 — jf3c06755_si_001.pdf [file jf3c06755_si_001.pdf]

Supporting information for

Heterologous naringenin production in the filamentous fungus *Penicillium rubens*

Bo Peng[a‡], Lin Dai[b‡], Riccardo Iacovelli[a], Arnold J. M. Driessen[b*], Kristina Haslinger[a*]

[a]*Chemical and Pharmaceutical Biology, Groningen Research Institute of Pharmacy, University of Groningen, Antonius Deusinglaan 1, 9713AV Groningen, The Netherlands*

[b]*Molecular Microbiology, Groningen Biomolecular Sciences and Biotechnology Institute, University of Groningen, Nijenborgh 7, 9747AG Groningen, The Netherlands*

‡Equal contribution, shared first authors

*Email: k.haslinger@rug.nl and a.j.m.driessen@rug.nl

Contents

Supporting information for 1

Heterologous naringenin production in the filamentous fungus *Penicillium rubens* 1

Supplementary materials 2

Supporting Tables 3

Supporting Figures 4

Supplementary materials

Sequences of synthetic genes

Pc4CL *(Petroselinum crispum*, GenBank accession number KX671122.1):

ATGGGTGACTGCGTTGCCCCGAAAGAGGATCTGATCTTCCGCAGCAAACTGCCGGACATTTACATTCCAAAGCATCTGCCGCT
GCATACCTATTGTTTTGAGAACATCAGCAAGGTTGGCGACAAGAGCTGTCTGATCAACGGCGCAACCGGCGAAACCTTTACCT
ACAGCCAGGTTGAGCTGCTGTCCCGTAAAGTTGCCAGCGGCCTGAACAAGCTGGGCATTCAACAAGGTGATACCATTATGCTG
CTGCTGCCGAACTCCCCGGAGTACTTTTTCGCTTTCCTGGGTGCGAGCTATCGCGGTGCAATCAGCACTATGGCGAACCCATT
CTTTACCAGCGCAGAAGTGATCAAGCAACTGAAAGCGAGCCAAGCGAAGCTGATTATCACCCAGGCATGCTATGTTGACAAGG
TTAAGGACTACGCAGCGGAGAAAAACATCCAGATCATTTGTATTGACGATGCACCGCAGGATTGCCTGCACTTTAGCAAGCTG
ATGGAAGCGGATGAGAGCGAAATGCCGGAAGTGGTTATTAACAGCGATGATGTGGTGGCACTGCCGTACAGCTCTGGCACCAC
CGGCCTGCCGAAAGGCGTTATGCTGACCCACAAGGGTCTGGTTACCAGCGTTGCACAACAGGTGGATGGTGATAACCCGAACC
TGTATATGCACTCCGAGGATGTTATGATCTGCATCCTGCCACTGTTCCATATCTATAGCCTGAACGCTGTTCTGTGTTGTGGT
CTGCGTGCGGGCGTTACCATTCTGATCATGCAAAAGTTCGACATTGTGCCGTTTCTGGAGCTGATTCAGAAGTATAAGGTTAC
CATTGGTCCGTTTGTTCCGCCGATCGTGCTGGCCATCGCGAAAAGCCCGGTTGTTGACAAGTACGACCTGTCTAGCGTGCGCA
CCGTTATGAGCGGTGCAGCGCCGCTGGGTAAAGAGCTGGAGGACGCTGTTCGTGCGAAATTCCCGAACGCGAAGCTGGGTCAA
GGCTATGGCATGACCGAAGCCGGTCCGGTTCTGGCGATGTGTCTGGCGTTCGCCAAAGAGCCGTATGAGATTAAGTCTGGCGC
ATGCGGTACCGTTGTGCGTAACGCCGAGATGAAAATCGTTGACCCAGAAACCAACGCGTCTCTGCCGCGTAACCAGCGTGGTG
AGATTTGCATCCGTGGTGATCAGATTATGAAAGGTTACCTGAACGACCCGGAAAGCACCCGCACCACCATCGACGAAGAGGGT
TGGCTGCACACCGGTGACATTGGTTTCATCGACGATGACGATGAACTGTTCATTGTTGATCGTCTGAAAGAAATCATTAAGTA
CAAAGGTTTTCAAGTTGCTCCGGCGGAGCTGGAAGCACTGCTGCTGACCCACCCGACCATCAGCGATGCCGCGGTGGTTCCGA
TGATTGACGAGAAAGCGGGTGAAGTGCCAGTGGCGTTTGTTGTGCGTACCAACGGTTTTACCACCACCGAAGAAGAAATCAAA
CAATTTGTGAGCAAACAGGTTGTGTTCTACAAACGTATCTTCCGCGTTTTCTTCGTTGACGCTATTCCGAAATCCCCGAGCGG
CAAGATTCTGCGTAAGGATCTGCGCGCTCGTATTGCGAGCGGCGACCTGCCGAAGTAA

PhCHS (*Petunia hybrida,* GenBank accession number KP284563.1): codon optimization for *E. coli*

ATGGTGACCGTGGAAGAATACCGTAAGGCGCAACGTGCGGAAGGCCCGGCGACCGTGATGGCGATTGGCACCGCGACCCCGAG
CAACTGCGTTGACCAGAGCACCTACCCGGATTTCTATTTTCGTATTACCAACAGCGAGCACAAAACCGACCTGAAGGAAAAAT
TCAAGCGTATGTGCGAGAAGAGCATGATTAAGAAACGTTACATGCACCTGACCGAGGAAATCCTGAAAGAGAACCCGAGCATG
TGCGAATATATGGCGCCGAGCCTGGACGCGCGTCAGGATATCGTGGTTGTGGAAGTGCCGAAACTGGGCAAAGAGGCGGCGCA
GAAAGCGATTAAGGAATGGGGTCAACCGAAAAGCAAGATCACCCACCTGGTTTTCTGCACCACCAGCGGCGTGGACATGCCGG
GTTGCGATTACCAACTGACCAAACTGCTGGGCCTGCGTCCGAGCGTTAAGCGTCTGATGATGTATCAGCAAGGTTGCTTTGCG
GGTGGCACCGTGCTGCGTCTGGCGAAAGATCTGGCGGAAAACAACAAGGGTGCGCGTGTTCTGGTTGTGTGCAGCGAGATTAC
CGCGGTGACCTTCCGTGGCCCGAACGACACCCACCTGGATAGCCTGGTTGGTCAGGCGCTGTTTGGTGATGGTGCGGGTGCGA
TCATTATCGGCAGCGATCCGATTCCGGGTGTTGAGCGTCCGCTGTTCGAACTGGTGAGCGCGGCGCAAACCCTGCTGCCGGAC
AGCCATGGTGCGATTGATGGTCACCTGCGTGAAGTTGGCCTGACCTTTCACCTGCTGAAAGACGTGCCGGGTCTGATTAGCAA
AAACATCGAGAAGAGCCTGGAGGAAGCGTTCAAGCCGCTGGGCATTAGCGACTGGAACAGCCTGTTTTGGATTGCGCACCCGG
GTGGCCCGGCGATTCTGGATCAAGTTGAAATCAAACTGGGCCTGAAGCCGGAGAAACTGAAGGCGACCCGTAACGTTCTGAGC
AACTACGGTAACATGAGCAGCGCGTGCGTGCTGTTTATCCTGGATGAAATGCGTAAAGCGAGCGCGAAAGAGGGTCTGGGTAC
CACCGGCGAGGGTCTGGAATGGGGTGTGCTGTTCGGCTTTGGTCCGGGCCTGACCGTGGAAACCGTTGTTCTGCATAGCGTTG
CGACCTAA

Supporting Tables

Table S1. Sequences of primers used in the study.

Primer	Sequence (5' to 3')	Application
lvl0_Pc4CL_F	TTGAAGACTTAATGGGTGACTGCGTTGCC	Building of pFL_0_1_Pc4CL
lvl0_Pc4CL_R	AAGAAGACAAAAGCTTACTTCGGCAGGTCGCCG	
lvl0_PhCHS_F	TTGAAGACTTAATGGTGACCGTGGAAGAATACCG	Building of pFL_0_2_PhCHS
lvl0_PhCHS_R	AAGAAGACAAAAGCTTAGGTCGCAACGCTATGCAGAAC	
lvl0_scr_F	AGTCAGTGAGCGAGGAAGC	Colony PCR for level 0 vectors
lvl0_scr_R	AATAGGCGTATCACGAGGC	
lvl1_scr_F	CACATTGCGGACGTTTTTAATGTACTG	Colony PCR for level 1 vectors
lvl1_scr_R	CCGCCAATATATCCTGTCAAACACTG	
Pc4CL_F	TGAATAGAAGACTCGGTGATGCAGCAAATAGCGACTGTTCGTTGCGGGGTCCGAACCCGCTCGGCAGCACCGGGCTCTCCCTACTATCCCTCGATAGCAGCTGCATTGGTCTGCCATTG	Amplification of donor DNA for in vivo HR of Pc4CL_ ergA_ PhCHS into *pen* locus
Pc4CL_R	CGCAGGGTTTGAGAACTCCGATCTTAAATCCAAGG	
ergA_F	GCAAGGTGCTATTCTAGGTAGGGTATGCCTAGCAATGCCATGATCTTATGACCTCAGTAATCTGACCTTGGATTTAAGATCGGAGTTCTCAAACCCTGCGTGCCTACCGCTCGTACCATGGG	
ergA_R	GACCGTCTATAACTCTGGATCCCCCGGGCTGCAG	
PhCHS_F	CTCTGCGTCCGTCCGTCTCTCCGCATGCCAGAAAGAGTCACCGGTCACTGTACAGAGCTCGAATTCCTGCAGCCCGGGGGATCCAGAGTTATAGACGGTCCGGCATAGGTAAGGAGAG	
PhCHS_R	GAATGCCGATTTGTCGGCAACGAGAGGTATGTCTAAGGTCTCGAGTTTAATTAGAATATTTACTAACAGCGTTTAGGAGCTCTTCCCCTAGCCAACTAGGTGCTTGGGATGTTCCATGG	
ergA_F2	TAGAAGACTCGGTGATGCAGCAAATAGCGACTGTTCGTTGCGGGGTCCGAACCCGCTCGGCAGCACCGGGCTCTCCCTACTATCCCTCGATAGCATACCGCTCGTACCATGGGTTG	Amplification of donor DNA for in vivo HR of ergA_ PhCHS into *pen* locus
sgRNA_F	AAAAAAGCACCGACTCGGTGCCACTTTTTCAAGTTGATAACGAACTAGTCTTATTTCAACTTGCTATGCTGTTTCCAGCATAGCTCTGAAAC	sgRNA targed on *pen* locus
sgRNA_R	ATGTAATACGACTCACTATA**GAACCAACATCATTAAGCAG**GTTTCAGAGCTATGCTGGAAA	
cPen_F	TGTTCGTAGAAGATCTGCCA	Colony PCR of Pc4CL integration
c4CL_R	CGCTTGGCTCGCTTTCAGTTGCTTGAT	
c4CL_F	ATCGACGATGACGATGAACTGT	Colony PCR of Pc4CL_ ergA_ PhCHS integration
cCHS_R	CCTCCAGGCTCTTCTCGATG	
cP40s_F	GAGTTATAGACGGTCCGGCATAGGTAAG	Colony PCR of PhCHS integration for 3F
cPen_R	ATTGCCAGTCTCACTATCCGATATGC	
cP40s_R	CCTTACCTATGCCGGACCGT	Colony PCR of ergA_PhCHS integration for 2F

Underlined bases: restriction site sequence; Bold: 20 bp target sequence of sgRNA; HR: homologous recombination.

Supporting Figures

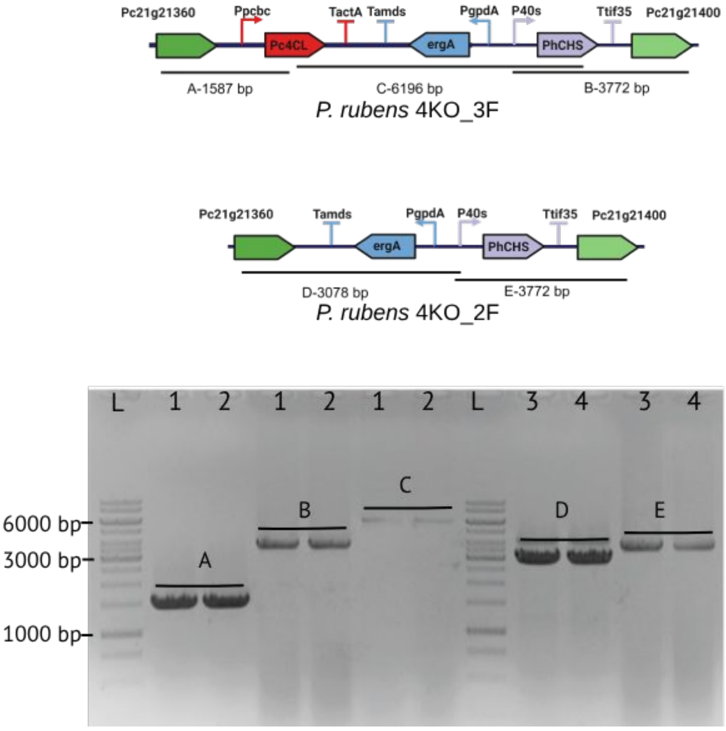

Figure S1. Colony PCR verification of integration of naringenin biosynthesis cluster into *P. rubens* 4xKO. Two colonies of the 3F and 2F variants were randomly selected and analyzed for the correct integration using the following PCR primer pairs: A) primers cPen_F and c4CL_R for the 3F variant; B) primers c4CL_F and cCHS_R for the 3F variant; C) primers cP40s_F and cPen_R for the 3F variant; D) primers cPen_F and cP40s_R for the 2F variant; and E) primers cPen_F and c4CL_R for the 2F variant. PCR amplified regions and expected size of amplicons are depicted on top. L: 1kb DNA ladder; 1, 2 are the PCR products of two 3F clones; 3, 4 are the PCR products of two 2F clones.

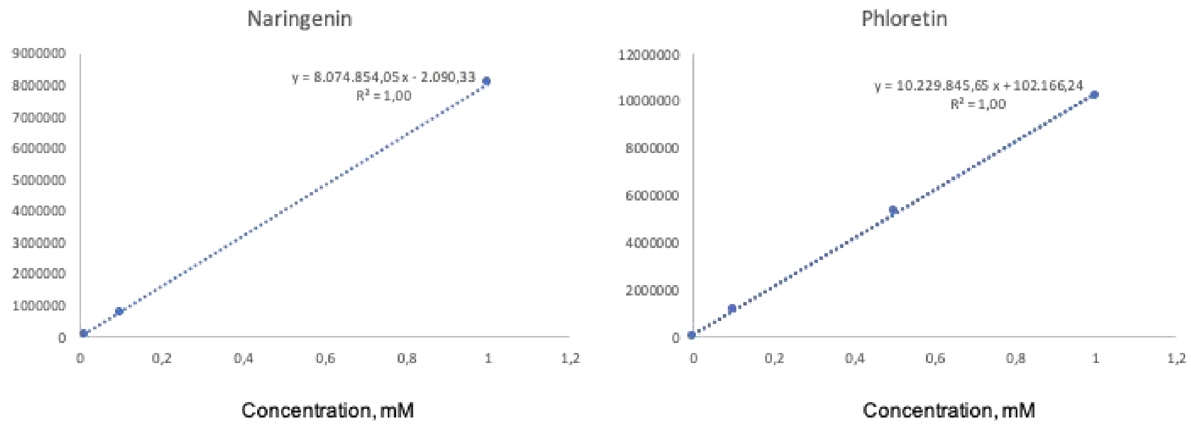

Figure S2. Calibration plot of naringenin and phloretin dissolved in DMSO and analyzed by HPLC. The compounds were detected at 288 nm and the analysis was performed as described in the method section. The range of calibration curve was 0.01 mM to 1 mM.

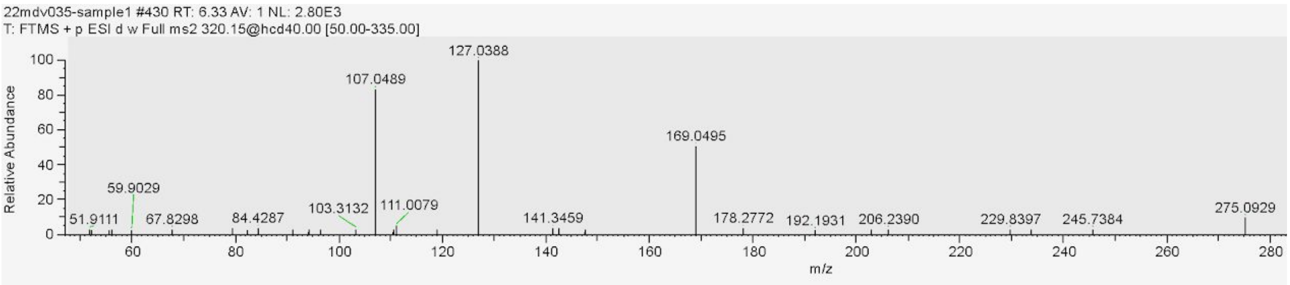

Figure S3. Product ion mass spectra (MS2) in high-resolution tandem MS of phloretin produced by *P. rubens* 4xKO-3F (275.0929 m/z [M+H]$^+$, RT=6.33 min).

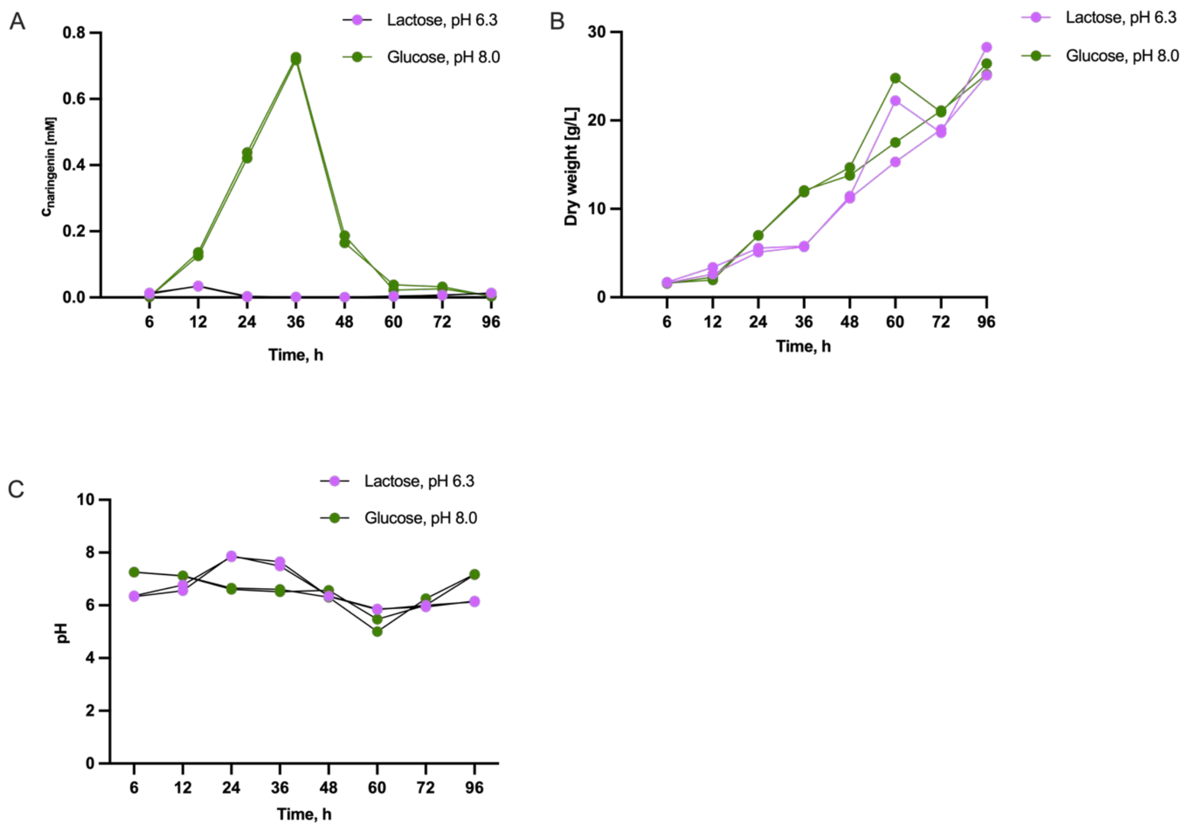

Figure S4. Time course of naringenin titer, dry weight, and medium pH of *P. rubens* 4xKO-3F in two different media: SMP with lactose as a carbon source, pH 6.3 (purple) and SMP with glucose as a carbon source, pH 8.0 (green). A) Naringenin titre over time, B) dry weight over time, C) pH over time. 1 mM *p*-coumaric acid was added after 1 d of cultivation and the experiment was performed with biological duplicates.

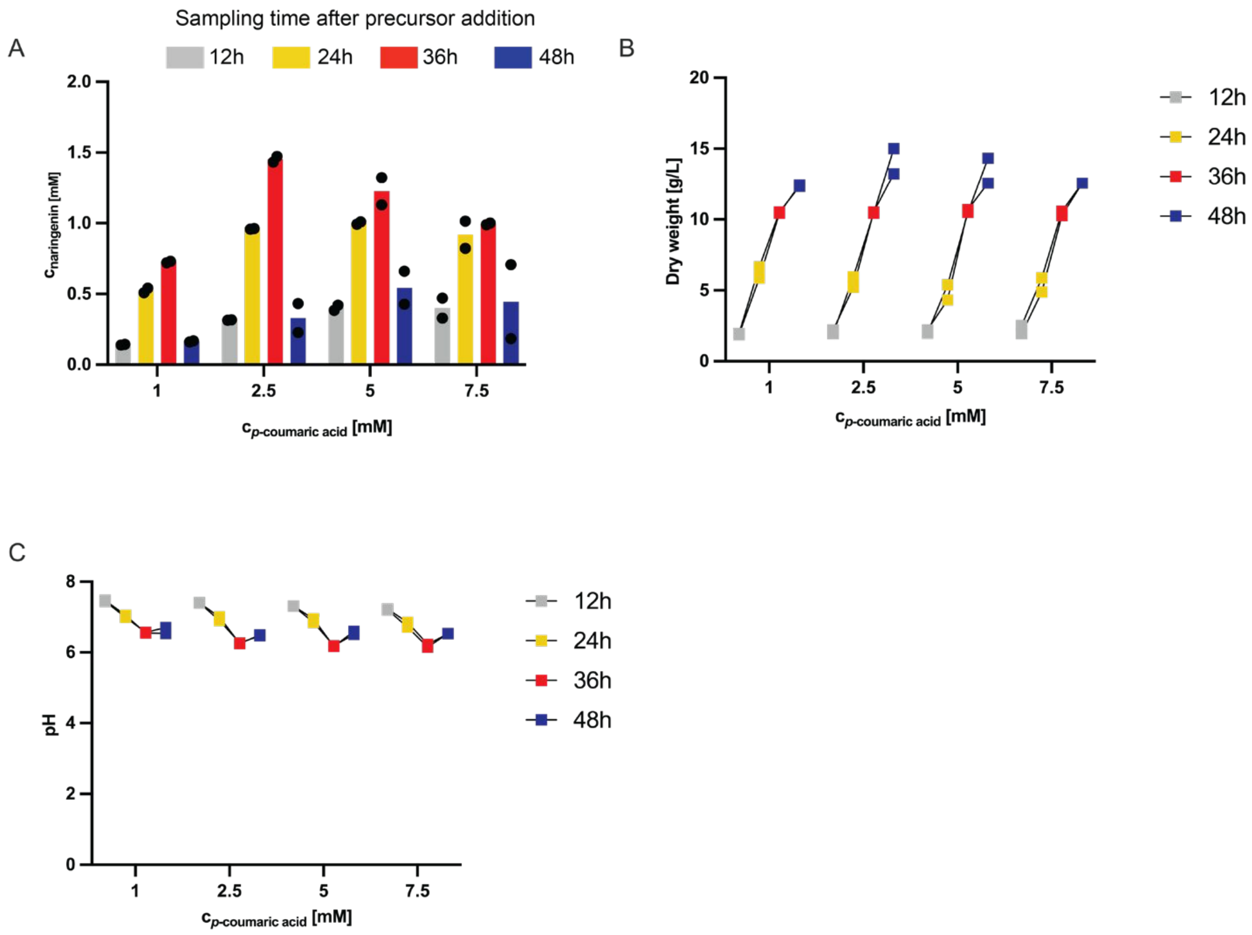

Figure S5. Time course of naringenin titer, dry weight, and medium pH during cultivation of *P. rubens* 4xKO-3F fed with different concentrations of *p*-coumaric acid. A) Naringenin titre over time, B) dry weight over time, C) pH over time in the presence of four different precursor concentrations. *p*-Coumaric acid was added after 24 h of cultivation. The experiment was performed in SMP media (glucose, pH 8.0) with biological duplicates.

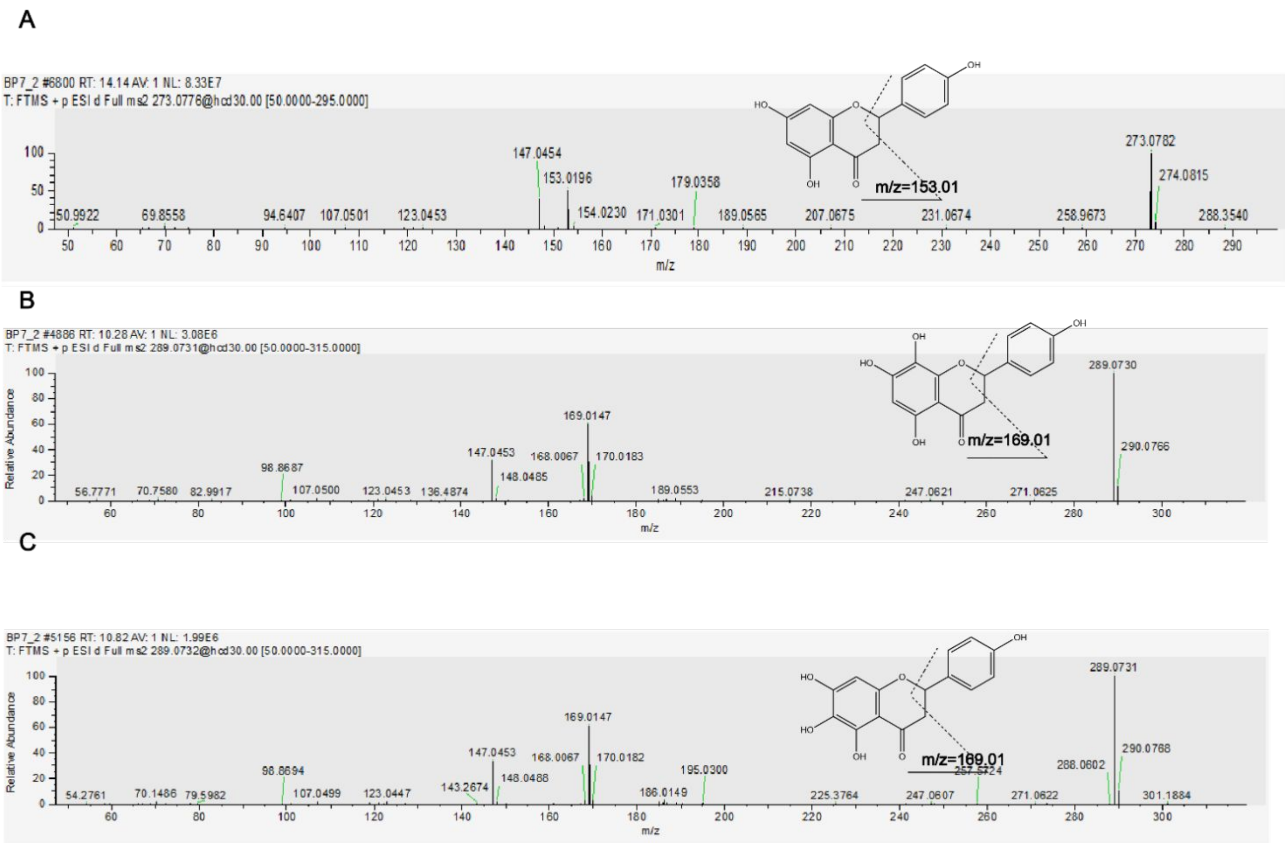

Figure S6. Product ion mass spectra (MS2) in high-resolution tandem MS of: A) naringenin (m/z 273.0776, RT=14.14 min), B) isocarthamidin (m/z 289.0728, RT=10.28), and C) carthamidin (m/z 289.0728, RT=10.82 min) extracted from the culture of *P. rubens* 4xKO in SMP medium (glucose, pH 8.0) supplemented with 1 mM naringenin.
